# Updates in Rhea – an expert curated resource of biochemical reactions

**DOI:** 10.1093/nar/gkw990

**Published:** 2016-10-26

**Authors:** Anne Morgat, Thierry Lombardot, Kristian B. Axelsen, Lucila Aimo, Anne Niknejad, Nevila Hyka-Nouspikel, Elisabeth Coudert, Monica Pozzato, Marco Pagni, Sébastien Moretti, Steven Rosanoff, Joseph Onwubiko, Lydie Bougueleret, Ioannis Xenarios, Nicole Redaschi, Alan Bridge

**Affiliations:** 1 Swiss-Prot Group, SIB Swiss Institute of Bioinformatics, CMU, 1 rue Michel-Servet, CH-1211 Geneva 4, Switzerland; 2 ERABLE team, INRIA Grenoble Rhône-Alpes, 655 avenue de l'Europe, F-38330 Montbonnot Saint-Martin, France; 3 Vital-IT, SIB Swiss Institute of Bioinformatics, Quartier Sorge, Bâtiment Génopode, CH-1015 Lausanne, Switzerland; 4 Department of Ecology and Evolution, Biophore, Evolutionary Bioinformatics group, University of Lausanne, CH-1015 Lausanne, Switzerland; 5 European Molecular Biology Laboratory, European Bioinformatics Institute (EMBL-EBI), Wellcome Trust Genome Campus, Hinxton, Cambridge CB10 1SD, UK; 6 University of Geneva, Department of Biochemistry, CH-1211 Geneva, Switzerland

## Abstract

Rhea (http://www.rhea-db.org) is a comprehensive and non-redundant resource of expert-curated biochemical reactions designed for the functional annotation of enzymes and the description of metabolic networks. Rhea describes enzyme-catalyzed reactions covering the IUBMB Enzyme Nomenclature list as well as additional reactions, including spontaneously occurring reactions, using entities from the ChEBI (Chemical Entities of Biological Interest) ontology of small molecules. Here we describe developments in Rhea since our last report in the database issue of Nucleic Acids Research. These include the first implementation of a simple hierarchical classification of reactions, improved coverage of the IUBMB Enzyme Nomenclature list and additional reactions through continuing expert curation, and the development of a new website to serve this improved dataset.

## INTRODUCTION

Rhea (http://www.rhea-db.org) is a comprehensive and non-redundant resource of expert-curated biochemical reactions designed for the functional annotation of enzymes and the description of metabolic networks (1). Rhea covers reactions of the hierarchical enzyme classification of the Enzyme Nomenclature committee of the IUBMB (hereafter referred to as the ‘EC’) (2,3) as represented by the ENZYME (4) and IntEnz (5) resources as well as additional enzymatic and transport reactions and spontaneously occurring reactions described in the literature.

Rhea reactions are defined by their participants and a specific reaction direction. Rhea represents small molecules and the functional groups of large macromolecules such as proteins using chemical entities from the ChEBI ontology (6), selecting the major microspecies (protonation state) for each ChEBI entity at an arbitrary pH of 7.3 and balancing all reactions for mass and charge accordingly. The curation of small molecule data is an integral part of the Rhea curation workflow, and Rhea curators have submitted thousands of new compounds to ChEBI during its development. Each set of reaction participants is associated to four potential directions: left to right (LR, =>), right to left (RL, <= ), bidirectional (BI, <=>) and undefined (UN, <?>), each with its own unique reaction identifier. Rhea reactions can be used to annotate the preferred direction ( =>, <=, <=>) of experimentally characterized enzymatic reactions in knowledgebases, to describe metabolic networks and derived models where reaction fluxes are not defined a priori (<?>), and to link knowledgebases and models. Knowledgebases that use Rhea for the annotation of enzyme and metabolite data include the SwissLipids knowledgebase for lipid biology (7), the EBI Enzyme portal (8), the MetaboLights repository of metabolomics data (9) and IntEnz (5). Resources that use Rhea for the annotation of metabolic networks and models include MetaNetX (10,11) and Microscope (12). Rhea also provides links to other metabolite and pathway databases such as KEGG (13), MetaCyc (14) and Reactome (15). More information about Rhea reactions and their use can be found in our previous publications (1,16).

In the following sections we summarize recent developments in Rhea since our last publication (1). These include the first implementation of a simple hierarchical classification of reactions, improved coverage of known enzymatic activities and additional reactions through continuing expert curation and the development of a new website to serve this enhanced dataset.

## CURRENT DEVELOPMENTS IN RHEA

### Rhea reaction classification

The EC uses a hierarchy of exactly four levels to classify enzyme activities according to a single representative reaction (referred to hereafter as the ‘representative reaction of the IUBMB’). Rhea (like other reaction databases) aims to provide complete coverage of all representative reactions of the IUBMB as well as other additional reactions described in the literature (whether or not these are related to an EC number).

To improve the annotation and classification of enzyme functions using Rhea we recently introduced a simple hierarchical reaction classification that covers representative reactions of the IUBMB as well as these additional reactions. This reaction classification uses ‘*is a*’ relationships to link ‘child’ reactions to their (more generic) ‘parent’ reactions, and allows any number of levels in order to facilitate the classification of extant and ancestral enzyme functions in ways that are meaningful to biologists. This may include the introduction of reaction classes that lie between existing levels of the EC and a finer grained classification of related reactions that are currently ‘compressed’ into the fourth level of the EC.

**Figure 1.**
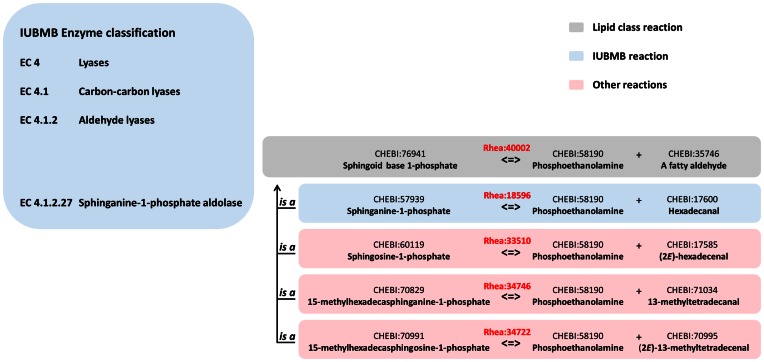
The Rhea reaction classification. The IUBMB enzyme classification (left) describes the enzyme activity sphinganine-1-phosphate aldolase (EC 4.1.2.27), which catalyzes the reaction Rhea:18596 (blue). This reaction, and other reactions including Rhea:33510, Rhea:34720 and Rhea:34746 (pink), are specific forms of the more generic reaction Rhea:40002 (grey), which lies between EC 4.1.2 and EC 4.1.2.27. Enzymes are omitted for the sake of clarity.

Figure 1 illustrates the use of the Rhea reaction hierarchy to classify the reactions associated with the enzyme activity sphinganine-1-phosphate aldolase (EC 4.1.2.27). The representative reaction of the IUBMB for this enzyme is ‘sphinganine 1-phosphate = phosphoethanolamine + palmitaldehyde’ (RHEA:18596), which is catalyzed by the enzyme SGPL1 of *Homo sapiens* as well as orthologous enzymes in other species (17). Sphinganine 1-phosphate is one member of a class of related phosphorylated sphingoid bases which vary in chain length, degree of saturation and branching. Additional related reactions (not mentioned by the EC reference list (3)) feature other members of this class such as sphingosine-1-phosphate (18) (RHEA:33510), the main phosphorylated sphingoid base in *H. sapiens*, as well as 15-methylhexadecasphinganine-1-phosphate (RHEA:34746) and 15-methylhexadecasphingosine-1-phosphate (RHEA:34722) (19), two phosphorylated sphingoid bases of *Caenorhabditis elegans* and the presumed substrates for the *C. elegans* SPGL1 homolog *spl-1*. The common ancestral function of *H. sapiens* SPGL1 and *C. elegans spl-1* could be summarized as ‘sphingoid-1-phosphate lyase’, which is a specialization of the ‘aldehyde-lyases’ (the third level of the EC classification, EC 4.1.2) and a generalization of ‘sphinganine-1-phosphate aldolase’ (the fourth and final level, EC 4.1.2.27). This common ancestral function therefore lies *between levels* of the EC. In Rhea a grouping reaction ‘a sphingoid 1-phosphate = a fatty aldehyde + phosphoethanolamine’ (RHEA:40002) was created using the newly defined grouping class of metabolite ‘a sphingoid 1-phosphate’ (CHEBI:76941) and existing metabolite classes. This grouping reaction links the representative reaction of the IUBMB to these additional related reactions. It can be used to annotate extant members of this orthologous group as well as ancestral functions in phylogenetic trees (20) at a greater level of precision than the generic ‘aldehyde lyase’ annotation EC 4.1.2.

The Rhea reaction classification was introduced in July 2016. The current release (release 75 of 30 July 30 2016) features over 600 expert-curated reaction relations. We are currently developing methods to calculate reaction relations for legacy data using curated relations in the ChEBI ontology (6) and computed measures of chemical structure and reaction similarity (21–23). We expect the number of reaction relations to increase significantly in the near future as we continue to check and validate calculated relations.

### Rhea content

Rhea has steadily grown since our last report through the expert curation of new chemical entities in ChEBI and reactions from peer-reviewed literature (see http://www.rhea-db.org/ statistics for details). At the time of writing, Rhea (release 75 of 30 July 30 2016) includes 9273 unique reactions (not considering directions) involving 8124 unique reaction participants, an increase of 2152 unique reactions and 2094 unique reaction participants since our last publication (release 53 of July 2014). Rhea covers over 94% of EC numbers with a defined reaction (4794 of 5124 EC numbers), and provides 4479 additional reactions. Many of these additional reactions were curated to support the generation of lipid libraries in the SwissLipids resource (7).

Rhea cites 8905 unique PubMed identifiers, an increase of 6142 since our last publication. This large increase in the amount of curated literature is the result of a concerted effort to map all enzymatic activities described in UniProtKB/Swiss-Prot to Rhea (including the representative reactions of the IUBMB and additional reactions) (see ‘Future directions’ section). During this process the existing literature from UniProtKB/Swiss-Prot was reviewed by Rhea curators and curated into Rhea where necessary.

### Rhea website

Since our last publication we have developed and deployed a new website at http://www.rhea-db.org. This website provides the same options for interactive and programmatic access as the previous version (1). Users can search for reaction and compound identifiers and names, EC numbers, UniProtKB/Swiss-Prot accession numbers, bibliographic citations and identifiers from external cross-referenced resources at http://www.rhea-db.org/advancedsearch. Reaction data can be downloaded in BioPax2 (24), RXN and RD (25) formats at http://www.rhea-db.org/download, which also provides access to the newly introduced reaction relations (described above) in tab-delimited form. Individual reactions can be bookmarked by adding the required identifier to the URL template http://www.rhea-db.org/reaction?id=, as in this example: http://www.rhea-db.org/reaction?id=10499. Reaction data in BioPax2 (24), RXN (25) and CMLReact (26) formats can also be obtained using RESTful web services at http://www.rhea-db.org/webservice.

## DISCUSSION

The UniProt consortium will use Rhea as a vocabulary for the annotation of enzymatic activities in UniProtKB from late 2017/early 2018 onward. To this end we continue to increase the coverage of Rhea through expert curation of new reactions, including representative reactions of the IUBMB and additional reactions described in peer reviewed literature. We also plan to develop an automated pipeline that assists Rhea reaction curation by identifying and prioritizing candidate reactions from the MetaNetX resource of genome scale metabolic models (10,11). More immediate developments are focused on a new RDF representation of Rhea data. This will be made available at a dedicated SPARQL endpoint to be hosted by the Vital-IT infrastructure (https://www.vital-it.ch/), which currently maintains a number of SPARQL endpoints such as http://sparql.uniprot.org/ and http://snorql.nextprot.org.
