# Updates in Rhea – an expert curated resource of biochemical reactions

**DOI:** 10.1093/nar/gkw1299

**Published:** 2016-12-15

**Authors:** Anne Morgat, Thierry Lombardot, Kristian B. Axelsen, Lucila Aimo, Anne Niknejad, Nevila Hyka-Nouspikel, Elisabeth Coudert, Monica Pozzato, Marco Pagni, Sébastien Moretti, Steven Rosanoff, Joseph Onwubiko, Lydie Bougueleret, Ioannis Xenarios, Nicole Redaschi, Alan Bridge

**Affiliations:** 1 Swiss-Prot Group, SIB Swiss Institute of Bioinformatics, CMU, 1 rue Michel-Servet, CH-1211 Geneva 4, Switzerland; 2 ERABLE team, INRIA Grenoble Rhône-Alpes, 655 avenue de l’Europe, F-38330 Montbonnot Saint-Martin, France; 3 Vital-IT, SIB Swiss Institute of Bioinformatics, Quartier Sorge, Bâtiment Génopode, CH-1015 Lausanne, Switzerland; 4 Department of Ecology and Evolution, Biophore, Evolutionary Bioinformatics group, University of Lausanne, CH-1015 Lausanne, Switzerland; 5 European Molecular Biology Laboratory, European Bioinformatics Institute (EMBL-EBI), Wellcome Trust Genome Campus, Hinxton, Cambridge CB10 1SD, UK; 6 University of Geneva, Department of Biochemistry, CH-1211 Geneva, Switzerland

*Nucl. Acids Res*. (2016) doi: 10.1093/nar/gkw990

The publishers would like to apologise for an error that was introduced into the title of this article. The correct title is not “Updates in rhea – an expert curated resource of biochemical reactions” but “Updates in Rhea – an expert curated resource of biochemical reactions”. This has been corrected above and in the original article.

